# Genetic Dissection of Vps13 Regulation in Yeast Using Disease Mutations from Human Orthologs

**DOI:** 10.3390/ijms22126200

**Published:** 2021-06-08

**Authors:** Jae-Sook Park, Nancy M. Hollingsworth, Aaron M. Neiman

**Affiliations:** Department of Biochemistry and Cell Biology, Stony Brook University, Stony Brook, NY 11794-5215, USA; jae-sook.park@stonybrook.edu (J.-S.P.); nancy.hollingsworth@stonybrook.edu (N.M.H.)

**Keywords:** chorea-acanthocytosis, ER-phagy, Vps13 adaptor, protein trafficking

## Abstract

The VPS13 family of proteins have emerged as key players in intracellular lipid transport and human health. Humans have four different *VPS13* orthologs, the dysfunction of which leads to different diseases. Yeast has a single *VPS13* gene, which encodes a protein that localizes to multiple different membrane contact sites. The yeast *vps13*Δ mutant is pleiotropic, exhibiting defects in sporulation, protein trafficking, endoplasmic reticulum (ER)-phagy and mitochondrial function. Non-null alleles resulting from missense mutations can be useful reagents for understanding the multiple functions of a gene. The exceptionally large size of Vps13 makes the identification of key residues challenging. As a means to identify critical residues in yeast Vps13, amino acid substitution mutations from *VPS13A*, *B*, *C* and *D*, associated with human disease, were introduced at the cognate positions of yeast *VPS13*, some of which created separation-of-function alleles. Phenotypic analyses of these mutants have revealed that the promotion of ER-phagy is a fourth, genetically separable role of *VPS13* and provide evidence that co-adaptors at the endosome mediate the activity of *VPS13* in vacuolar sorting.

## 1. Introduction

The VPS13 proteins are a novel family of lipid transfer proteins important for human health [1]. Humans express four different *VPS13* paralogs, designated A through D. Mutations in *VPS13A*, *VPS13B*, *VPS13C* and *VPS13D* are associated with the neurodegenerative disorder Chorea-Acanthocytosis (ChAc), Cohen Syndrome, Parkinson’s Disease, and a form of cerebellar ataxia, respectively [2,3,4,5,6,7]. A detailed understanding of the localization and activity of this protein family is essential for developing treatments for these various conditions.

Much of what is known about the molecular function of the VPS13 family of proteins comes from studies in the budding yeast, *Saccharomyces cerevisiae*, which contains a single *VPS13* gene [8]. Yeast *VPS13* is required for a variety of different cellular processes, including (1) the proper trafficking of the vacuolar protein carboxypeptidase Y (CPY) through the Golgi and endosomal compartments to the vacuole [9]. (2) Sporulation: the process by which the haploid products of meiosis are packaged into spores [10]. *VPS13* is necessary for proper formation of the prospore membranes that engulf the haploid nuclei [11]. (3) Mitochondrial homeostasis: *VPS13* becomes essential for growth when lipid transfer by the ER-mitochondrion encounter site (ERMES) complex is disrupted [12,13]. (4) ER-phagy: *VPS13* is required for the selective autophagy of the cortical ER [14].

Vps13 localizes to a number of different membrane contact sites within the yeast cell [12,13,15]. Membrane contact sites are connections between the limiting membranes of different organelles and serve as a point of transfer for lipids and metabolites between organelles [16,17]. Vps13 localization varies with growth conditions. In cells grown in glucose, Vps13 is predominantly on endosomes and at endosome–mitochondrion contact sites [12,13]. In acetate medium, Vps13 primarily localizes to nuclear–vacuole junctions [12,13]. Vps13 is present along the prospore membranes in sporulating cells, consistent with its role in prospore membrane morphogenesis [11]. Structural and biochemical studies indicate that Vps13 present at contact sites mediates lipid transfer between organelles through a hydrophobic channel that spans the distance between the different organellar membranes [18,19,20]. The pleiotropic phenotypes observed in the absence of *VPS13* have been explained by the simultaneous disruption of lipid transfer between different pairs of organelles [12,13,19].

The localization of Vps13 to different contact sites is controlled by binding to different organelle-specific partner proteins, termed adaptors [21]. Different Vps13 adaptors compete for binding to a region termed the Vps13 adaptor binding domain (VAB) [21] (Figure 2A). For example, *Ypt35*, a lipid-binding protein found on endosomal and vacuolar membranes, binds to the VAB domain and is necessary for Vps13 recruitment to these membranes [21]. Similarly, Spo71 is a sporulation-specific protein that recruits Vps13 to prospore membranes through interaction with the VAB domain [21,22].

The paradigm for Vps13 function as a lipid transfer protein at different membrane contact sites likely applies to human VPS13 protein family members as well. VPS13A, C and D localize to various membrane contact sites in cultured human cells, including ER-mitochondrion, ER-endosome, and ER-lipid droplets. Furthermore, VPS13D mediates lipid transfer in vitro [18,23,24,25]. Proper localization of the VPS13 paralogs is important in human cells, since mutations in the VAB domains of *VPS13A* and *VPS13D* are associated with neurological defects and a missense mutation in the *VPS13A* VAB domain abolishes localization to lipid droplets [2,26,27]. These results suggest that, like yeast, human VPS13 family proteins are localized to membrane contact sites through interaction with adaptor proteins via the VAB domain to mediate lipid transfer between organelles.

Yeast Vps13 is an extremely large protein containing 3144 amino acids with multiple distinct domains (Figure 2A). Determining which parts of the protein are necessary for different Vps13 functions is therefore a daunting task. One approach used to identify the key amino acids necessary for one or more Vps13 functions takes advantage of the numerous mutations found in the human *VPS13* paralogs that are associated with human diseases. Because of the strong conservation of VPS13 family proteins, cognate mutations can be introduced into yeast *VPS13* and the resulting mutants characterized for defects in various processes [13,28,29]. This approach previously revealed that missense mutations in *VPS13A* found in ChAc patients are specifically defective in the mitochondrial function of yeast *VPS13* [13].

This work systematically characterizes the yeast phenotypes of a set of missense mutations contained in human *VPS13* paralogs that are associated with disease. While no phenotypes were observed for any of the disease mutations in *VPS13B* or *VPS13C*, a *VPS13D* cognate mutant (N2428S) was discovered that affects both CPY sorting and ER-phagy. In addition, the Vps13^N2428S^ protein fails to localize to endosomes and an intragenic suppressor mutation that restores localization of Vps13^N2428S^ to the endosome restored CPY sorting but not ER-phagy. These results supply evidence of a link between endosomal localization of Vps13 and vacuolar protein sorting and demonstrate that the promotion of ER-phagy is a distinct function of yeast *VPS13*.

## 2. Results

### 2.1. VPS13D Cognate Mutants, but Not VPS13B or C, Disrupt a Subset of VPS13 Functions in Yeast

**Figure 1:**
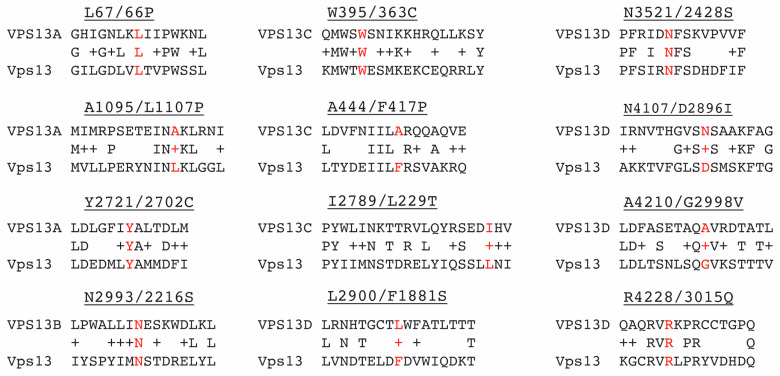
Alignments of regions of different human VPS13 paralogs and yeast Vps13. Labels have the format “Mammalian amino acid and position/yeast cognate amino acid and position, ending with the amino acid in the mammalian gene mutant”. The cognate amino acids are highlighted in red. All the alignments were produced by BLAST search except for the regions around VPS13B N2993 and VPS13D L2900, which were based on the VAB repeat sequences defined in [21].

**Figure 2:**
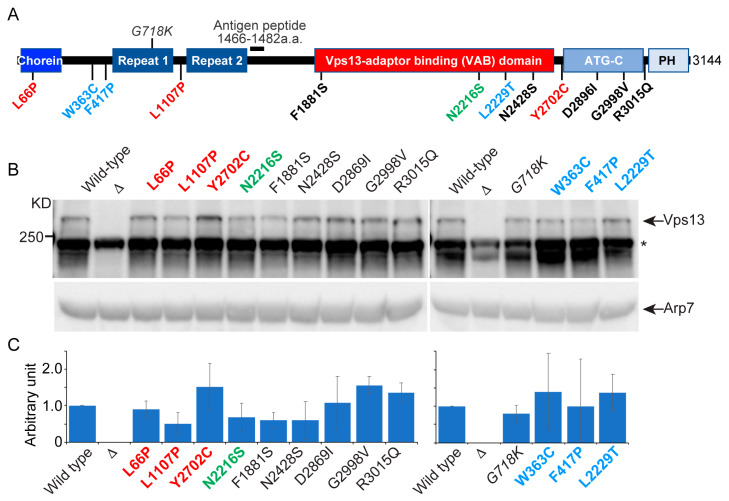
Steady-state levels of yeast Vps13 proteins carrying cognate mutations of various human *VPS13* paralogs (**A**) The position of *VPS13* cognate mutations with respect to domains of the Vps13 protein. Domains are not drawn to scale. Mutations from the different human *VPS13* paralogs are color coded: *VPS13A*, red; *VPS13B*, green; *VPS13C*, blue; *VPS13D*, black. *G718K* is a suppressor mutation identified in yeast [13]. The black bar indicates the location of the peptide used to generate the Vps13 antibody (a.a. = amino acid). (**B**) Immunoblot analysis. Protein extracts from the following strains: wild type (BY4741), *vps13*Δ (KO1), *vps13-L66P* (RP201), *vps13-L1107P* (RP202), *vps13-Y2702C* (RP203), *vps13-N2216S* (JSP770), *vps13-F1881S* (JSP786), *vps13-N2428S* (JSP757), *vps13-D2869I* (JSP787), *vps13-G2998V* (JSP788), *vps13-R3015Q* (JSP758), *vps13-G718K* (JSP549), *vps13-W363C* (RP206), *vps13-F417P* (RP207), *vps13-L2229T* (JSP729) were probed with anti-Vps13 and anti-Arp7 antibodies. The white line indicates two different blots. The asterisk indicates a cross-reacting band. Full blot is shown in Supplementary Figure S1. (**C**) Quantification of Vps13 mutant proteins relative to wild-type Vps13 ([Vps13-X/Arp7]/[Vps13/Arp7]). Graphs show the average of three independent experiments. Error bars indicate one standard deviation.

Pairwise alignments between each of the human VPS13 orthologs and yeast Vps13 identified cognate residues in the yeast protein for disease-associated missense mutations (Figure 1). A residue was considered a likely cognate if it aligned in a region of strong sequence conservation. The amino acid substitutions caused by the mutations are distributed throughout the Vps13 protein, including the conserved chorein domain in the N-terminus, the VAB and the ATG-C domains (Figure 2A). A two-step CRISPR-Cas9 strategy was used to introduce mutations into *VPS13* on the chromosome [13]. First, the *Saccharomyces kluyveri HIS3* gene (*SkHIS3*) was substituted for the *VPS13* codon to be mutated. Next, cells were transformed with a *LEU2* replicating plasmid carrying genes for both the Cas9 endonuclease and a guide RNA targeting *SkHIS3*. Included also was a rescue oligonucleotide containing the desired missense mutation and *VPS13* sequences flanking *SkHIS3*. Only transformants that have repaired the double strand break and destroyed the cut site are able to grow [30]. Leu^+^ transformants were screened for the loss of *SkHIS3* on a medium lacking histidine. This phenotype could result either from non-homologous end-joining that disrupted the *SkHIS3* gene or homologous recombination using the rescue oligonucleotide as the template [30]. These two possibilities were distinguished by amplifying the region using the polymerase chain reaction (PCR) and DNA sequencing to confirm the loss of *SkHIS3* and the presence of the desired mutations.

To determine whether mutant phenotypes were due to Vps13 instability, steady state protein levels were assessed by immunoblot analysis. Previously, Vps13 was detected by epitope tagging the protein, a laborious process because only internal tags are fully functional and the large size of the gene makes recombinant manipulations difficult [12,13]. A polyclonal antibody was therefore generated using a peptide in the amino-terminal half of the protein to allow detection of endogenous Vps13 (Figure 2A). The specificity of the Vps13 antibody was confirmed by showing it recognized a protein with a molecular weight > 250 kD (the predicted molecular weight for Vps13 is 350 kD) that was absent in *vps13*Δ (Figure 2B). Arp7 protein was used as a loading control [31]. The ratio of the intensity of the Vps13 and Arp7 bands in each mutant strain relative to the *VPS13* strain was used to quantify relative Vps13 protein levels. The Vps13-F1881S protein exhibited a ~2-fold reduction in protein levels but was phenotypically wild type in every assay (Figure 2C, Table 1). Therefore, defects displayed by *vps13* alleles encoding proteins with ≥0.5 Vps13 levels are most likely not due to insufficient amounts of protein.

**Figure 3:**
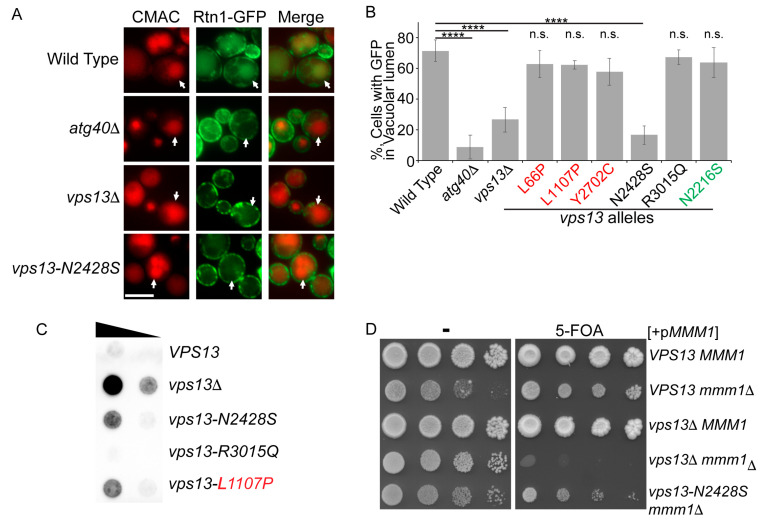
Characterization of the *vps13-N2428S* allele. (**A**) ER-phagy: cells at mid-log phase were treated with rapamycin for 16 h and stained with the dye CMAC to indicate the position of the vacuole. ER-phagy results in the co-localization of the cortical ER protein, Rtn1-GFP, with the vacuole. Wild type (GCY5), *atg40*Δ (JSP845), *vps13*Δ (JSP846) and *vps13-N2428S* (JSP851). Arrows highlight the positions of the vacuole in representative cells as shown by CMAC fluorescence (in red). Scale bar = 5 µm. (**B**) The fraction of cells exhibiting Rtn1-GFP fluorescence in the vacuolar lumen after rapamycin treatment in different *VPS13* mutants; strains as in (**A**), plus *vps13-L66P* (JSP847), *vps13-L1107P* (JSP848), *vps13-Y2702C* (JSP849), *vps13-R3015Q* (JSP852), and *vps13-N2216S* (JSP854). Numbers are the averages of at least three independent experiments with over 150 cells per strain scored in each experiment. Error bars indicate one standard deviation. **** indicates *p* < 0.0001, Student’s *t*-test, n.s. not significant. (**C**) CPY secretion assay. Cells were grown to exponential phase in liquid YPD, adjusted to equalize cell numbers between cultures and ten-fold dilutions were spotted onto SD complete medium. A nitrocellulose membrane was placed over the cells and the plates were incubated overnight at 30 °C. The filter was then rinsed and probed with anti-CPY antibodies. Strains used were wild type (BY4741), *vps13*Δ (KO1), *vps13-N2428S* (JSP757), *vps13-R3015Q* (JSP758), and *vps13-L1107P* (RP202). (**D**) Synthetic lethality assay with *mmm1*Δ. Strains of the indicated genotype carrying pRS316-*MMM1* were grown overnight at 30 °C in YPD medium; wild type (BY4741), *mmm1*Δ (JSP441), *vps13*Δ (KO1), *vps13*Δ *mmm1*Δ (JSP443) and *vps13-N2428S mmm1*Δ (JSP759). Tenfold serial dilutions were spotted onto SD complete medium with or without 5-FOA and grown at 30 °C for 4 days. Color code for allele designations as in Figure 2.

Four assays were used to examine the effects of *VPS13* cognate mutations on different processes in yeast. (1) Protein sorting: CPY is a protein that normally transits from the ER to the vacuole. Defects in protein sorting at the Golgi result in the secretion of CPY into the medium [9]. Improper sorting can be detected by placing a nitrocellulose filter on a patch of cells, allowing the cells to grow, then removing the filter and probing it with CPY antibodies to detect CPY secreted onto the filter [32]. (2) Sporulation: diploid cells hemizygous for the cognate mutations (*vps13-x*/*vps13*Δ) were placed onto sporulation medium, incubated at 30 °C for two days and then examined by light microscopy for the presence of asci. (3) Mitochondrial homeostasis: *VPS13* is essential in the absence of the ERMES complex [12,13]. Haploids containing *mmm1*Δ (a component of ERMES) and *vps13*Δ are viable in the presence of a *URA3 MMM1* replicating plasmid but fail to grow on medium containing 5-fluororotic acid (5-FOA), which selects for loss of the plasmid [33]. Haploids containing *vps13-x mmm1*Δ/p*MMM1 URA3* were tested for 5-FOA resistance. Failure to grow indicated a defect in mitochondrial homeostasis. (4) ER-phagy, the selective autophagy of the cortical ER in response to starvation [14]. ER-phagy was detected using a green fluorescent protein (GFP) fusion to a cortical ER protein called Rtn1. After starvation was induced by incubation for several hours in medium supplemented with rapamycin, cells were examined for the presence of Rtn1-GFP in the vacuole (Figure 3A) [14]. *ATG40* is required for ER-phagy and the absence of Rtn1-GFP in the vacuole after starvation in the *atg40*Δ haploid was used as a control in this assay [34].

None of the missense mutations created mutants defective in all of these processes (Table 1). Several mutants, including all of the cognate mutants from *VPS13B* and *C*, and *D* (with one exception, see below), exhibited wild-type phenotypes for all four *VPS13* functions. Previous work showed that the *VPS13A* cognate mutant, L66P is specifically defective in mitochondrial homeostasis, while L1107P is defective in both CPY sorting and mitochondrial homoeostasis, but not sporulation [13]. Neither mutant exhibited defects in ER-phagy, further underscoring their specificity (Table 1; Figure 3B). These results reinforce the idea that a defect in mitochondrial function is a common feature of *VPS13A* alleles when they are expressed in yeast, suggesting that a mitochondrial role may be important for *VPS13A* function in human cells.

The *VPS13D* cognate mutant, *vps13-N2428S*, displayed a novel phenotype. It sporulated like wild type and exhibited a mild mitochondrial phenotype but was partially defective in both CPY sorting and ER-phagy (Table 1 and Table 2, Figure 3). The ER-phagy mutant phenotype does not correlate with a CPY sorting defect, as *vps13-L1107P* is similarly defective for CPY sorting, but not for ER-phagy (Table 1, Figure 3B,C). Furthermore, Vps13-L1107P and Vps13-N2428S were produced at comparable levels (Figure 2B,C), indicating that decreased protein levels cannot account for the ER-phagy defect in the *vps13-N2428S* strain, though a reduced protein level may explain the modest mitochondrial phenotype (Figure 3D).

### 2.2. Vps13^N2428S^ Localizes Normally to the Prospore Membrane

**Figure 4:**
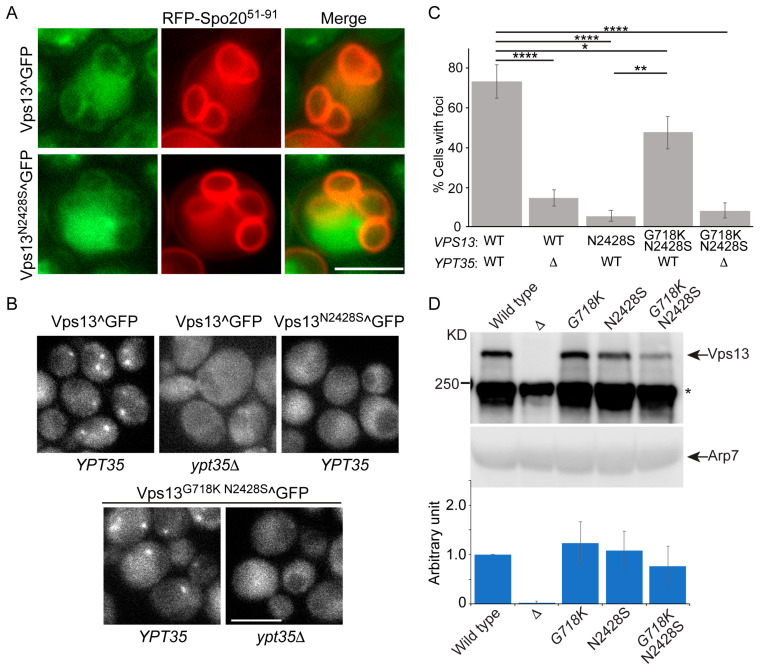
Localization of Vps13^N2428S^^GFP under various conditions. (**A**) Sporulation: *VPS13^GFP/vps13*Δ (JSYD4) and *vps13-N2428S^GFP/vps13*Δ (JSYD12) diploids carrying the prospore membrane marker RFP-Spo20^51-91^ (pRS426-R20) were transferred to SPO medium and proteins were visualized by fluorescence microscopy [38]. Representative cells are shown from over 100 cells with prospore membranes. (**B**) *VPS13^GFP* (JSP497), *VPS13^GFP ypt35*Δ (JSP861), *vps13-N2428S^GFP* (JSP816), *vps13-G718K N2428S^GFP* (JSP832), or *vps13-G718K N2428S^GFP ypt35*Δ (JSP856) were grown to log-phase in 2% glucose and then analyzed for GFP fluorescence. Scale bars = 5 µm. (**C**) Quantification of GFP foci in vegetative cells in glucose. Percentages are the average of at least three independent experiments with over 130 cells per strain scored in each experiment. The error bars indicate one standard deviation. **** indicates difference is significant to *p* < 0.0001, ** *p* < 0.01, * *p* < 0.05 by Student’s *t*-test. WT = wild type. (**D**) Western blot showing levels of the wild type and mutant GFP fusion proteins. Lower panel, levels of the mutant proteins relative to wild type, average of three experiments. Full blot is shown in Supplementary Figure S2.

The N2428S mutation lies in the VAB that mediates binding to adaptor proteins and this mutation was previously reported to cause a general defect in adaptor binding, including the prospore membrane adaptor, Spo71 [28]. A failure of Vps13 to localize to the prospore membrane should prevent sporulation, however in our strains, the *vps13-N2428S/vps13*Δ diploid sporulated as efficiently as *VPS13/vps13*Δ (Table 2), suggesting that the mutant protein is present on prospore membranes. To directly test this hypothesis, the Vps13^N2428S^ protein was visualized within the cell by inserting GFP internally at amino acid 1360 (Vps13^N2428S^^GFP), a position that does not disrupt Vps13 function [13]. A plasmid encoding a fusion of red fluorescent protein (RFP) to the phosphatidic acid-binding domain of Spo20 was used as a marker for prospore membranes [37]. Vps13^N2428S^^GFP localized to the prospore membranes similarly to the wild-type protein (Figure 4A), suggesting that substituting S for N2428 does not disrupt binding to Spo71 during sporulation.

### 2.3. An Intragenic Mutation Restores Endosomal Localization to Vps13^N2428S^ and Partially Suppresses the CPY Sorting Defect

**Figure 5:**
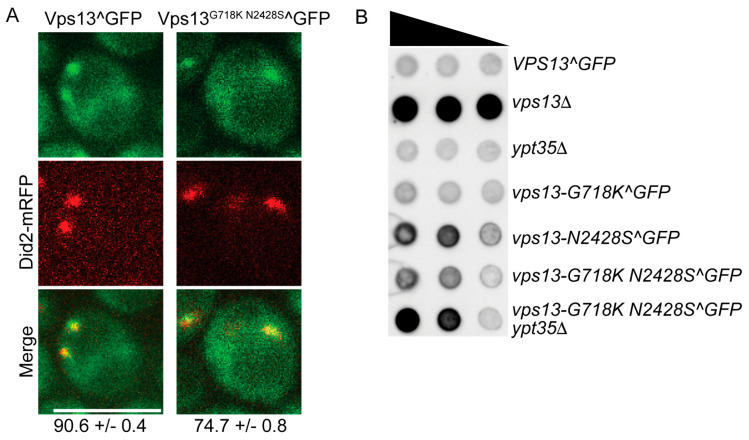
The intragenic *vps13-G718K* mutation restores endosomal localization of Vps13^G718K N2428S^ and promotes CPY sorting. (**A**) Colocalization of Vps13^GFP proteins with endosomes. Strains carrying *VPS13^GFP* (JSP512) or *vps13-G718K N2428S^GFP* (JSP864) containing the endosomal marker, *DID2-mRFP*, were grown in SD complete medium and collected at mid-log phase for microscopy [39]. Scale bars = 5 µm. Numbers at the bottom indicate the percentage of Vps13 foci that co-localized with the Did2-mRFP marker (±one standard deviation). Two experiments with at least 100 foci were scored for each experiment). (**B**) CPY sorting. The indicated strains were assayed for CPY sorting as described in Figure 4 except that 4-fold serial dilutions were used; *VPS13*^GFP (JSP497), *vps13*Δ (KO1), *ypt35*Δ (KO6), *vps13-G718K^GFP* (JSP531), *vps13-N2428S^GFP* (JSP816), *vps13-G718K N2428S^GFP* (JSP832) and *vps13-G718K N2428S^GFP ypt35*Δ (JSP856).

In wild-type cells, Vps13 localization to the endosome is dependent upon the adaptor protein encoded by *YPT35* [21] (Figure 4B). In contrast, the *N2428S* substitution abolishes Vps13 localization to the endosome, consistent with an earlier report that interaction with *Ypt35* is disrupted by this VAB mutation [28] (Figure 4B,C). Previous work has shown that the *vps13-L66P* mutant exhibits mitochondrial homeostasis defects and displays loss of Vps13 from ER-mitochondrion contact sites and a redistribution of the protein towards nuclear–vacuole junctions [13]. Introduction of a second mutation (G718K) into the *vps13-L66P* allele restored a more wild-type distribution of the protein to the mitochondrion as well as its mitochondrial function [13]. Whether the G718K mutation could similarly restore the localization of Vps13^N2428S^ to the endosome was examined. Indeed, the mutant protein encoded by *vps13-G718K N2428S^GFP* restored the cytoplasmic puncta that were missing in *vps13-N2428S^GFP* and these foci were dependent upon *YPT35* (Figure 4B,C). This restoration of function was not due to changes in protein level, as the levels of all the GFP fusions were similar to wild type (Figure 4D). The organellar identity of the Vps13^G718K N2428S^ foci as the endosome was confirmed by co-localization with the endosomal marker Did2-mRFP (Figure 5A). Thus, the G718K mutation restores the *YPT35*-dependent localization of the Vps13^N2428S^ protein to the endosome. Furthermore, this relocalization of the Vps13^G718K N2428S^ to the endosome partially suppresses the CPY sorting defect (Figure 5B).

Although *YPT35* is required for Vps13 localization to the endosome, deletion of *YPT35* does not create a defect in CPY sorting, raising the possibility that endosomal localization is not important for vacuolar sorting (Figure 4B and Figure 5B). The *vps13-N2428S* phenotypes, however, argue instead that endosomal localization is important: both that restoring localization to the endosome with the G718K mutation improves CPY sorting and that the *ypt35*Δ in fact increases CPY secretion in the double mutant (Figure 5B) (see Discussion).

### 2.4. The vps13-G718K Mutant Alone Is Defective in ER-phagy

**Figure 6:**
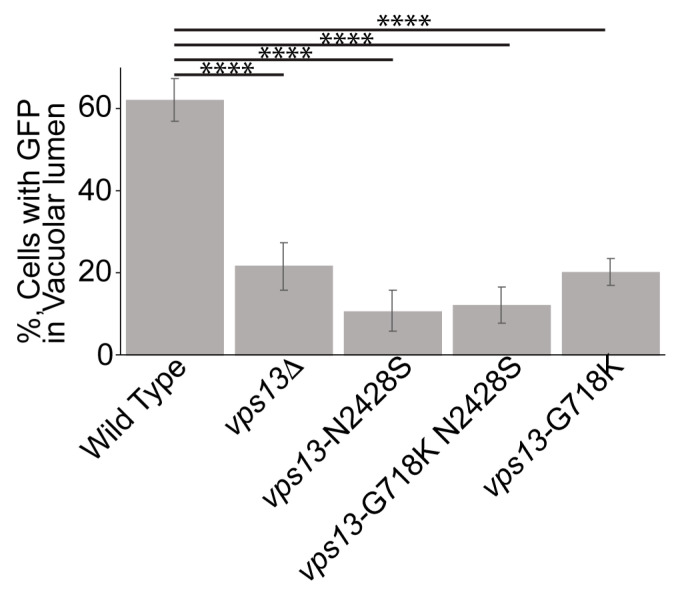
The *vps13-G718K* mutant is defective in ER-phagy. Quantification of ER-phagy in different strains: wild type (GCY5), *vps13*Δ (JSP846), *vps13-N2428S* (JSP851), *vps13-G718K N2428S* (JSP881), and *vps13-G718K* (JSP883). Percentages shown are the averages of at least four independent experiments and over 150 cells per strain were scored in each experiment. Error bars are one standard deviation. **** indicates comparison to wild type, *p* < 0.0001.

To see whether the G718K suppression of *vps13-N2428S* is specific to the endosomal function of *VPS13*, ER-phagy was assessed in double mutant. However no conclusions could be made due to the unexpected discovery that *vps13-G718K* itself reduces ER-phagy (Figure 6). As *vps13-G718K* is wild-type for CPY sorting (Figure 5B) this observation provides further evidence that ER-phagy is unrelated to the role of Vps13 in vacuolar sorting.

## 3. Discussion

While many human disease alleles of *VPS13* contain indels or splice site mutations, there are also missense mutations that have the potential to be non-null alleles and a source of useful genetic tools for dissection of *VPS13* function. Indeed, all of the cognate mutants that exhibited phenotypes were defective in only a subset of *VPS13*-dependent processes. Interestingly, all of the *VPS13B* and *C* cognate mutants were completely functional in yeast. One explanation is that the substituted amino acid residues were incorrectly deemed cognates to those of the human proteins. Alternatively, the mutations may interfere with a function of the human protein that is not shared with the yeast protein, or in a yeast process that has not yet been identified. It is noteworthy that *VPS13A* mutants more frequently displayed phenotypes in yeast. VPS13A has the highest degree of relatedness to yeast Vps13, which might reflect greater conservation of function [40].

Our analysis of the phenotypes of these human alleles provides an important insight into the activities and regulation of yeast Vps13. For example, the fact that *vps13-L1107P* is defective in CPY sorting and mitochondrial homeostasis but is functional for ER-phagy shows that the role of Vps13 in ER-phagy is not tied to these other functions. More strikingly, *vps13-G718K* is defective in ER-phagy but wild-type for vacuolar sorting, mitochondrial function, and sporulation (Figure 6; [13]; J.S.P., unpublished obs.). The phenotypes of *vps13-G718K* demonstrate that ER-phagy is a genetically separable function of *VPS13*.

The G718K mutation is one of 15 different mutations in *VPS13* that suppress the slow growth exhibited by ERMES mutants [12,13,41]. Because deletion of *VPS13* is synthetically lethal with ERMES mutants and the suppressor mutants alone had previously exhibited no mutant phenotypes, these *VPS13* suppressors appeared at first glance to be gain-of-function mutants. However gain-of-function mutants are usually rare and highly specific, while these *VPS13* suppressors result from different amino acid substitutions that are distributed throughout a 1600 amino acid region of the protein. The variety of different amino acid substitutions that exhibit the suppressor phenotype, as well as the ease with which they are found, has led to the suggestion that suppression is due to the loss of a negative regulatory function of Vps13 [12]. Our discovery that the G718K mutant is not completely wild-type, but is, in fact, defective in ER-phagy, argues against this hypothesis.

What is common to the intragenic suppression of both the *vps13-L66P* mitochondrial phenotype and the CPY sorting defect of *vps13-N2428S* by G718K is that G718K restores the localization of the mutant proteins to their sites of action. One explanation for this effect would be if the suppressor mutations released the protein from some location—potentially a localization important for ER-phagy—thereby making more Vps13 available for recruitment to other contact sites. It is worth noting that the mutants suppressed by G718K are both based on disease-causing alleles. It will be of interest to learn if the cognate mutations to G718K in the human genes suppress the phenotypes of *VPS13A* or *VPS13D* mutations in mammalian cells. Conceivably, a pharmacological agent that had the same effect as the suppressor mutations could have therapeutic value, at least for patients carrying these specific alleles.

Here we characterized one allele, *vps13-N2428S*, which shows defects in CPY sorting and ER-phagy, but not in sporulation or mitochondrial function. In an earlier study, this allele was also found to have CPY sorting defects, as well as reduced interactions with all of the Vps13 adaptor proteins as determined by co-immunoprecipitation experiments [28]. However, when sporulation was measured directly in our experiments, *vps13-N2428S* sporulated as well as wild type (Table 2). These results suggest that the Vps13^N2428S^ mutant protein has reduced affinity for the adaptors, such that the interaction is disrupted under immunoprecipitation conditions but retains sufficient affinity to support function in vivo. In agreement with the earlier study, we found that the *N2428S* mutation caused a release of Vps13 from the endosome, similar to *ypt35*Δ. The G718K mutation, acts as an intragenic suppressor to restore endosomal localization and partially suppress the CPY sorting defect of the *vps13-N2428S* cells.

These results present a paradox, however. The correlation of endosomal localization with a CPY sorting defect in *vps13-N2428S* suggests that endosomal localization is important for proper CPY sorting. However, CPY is sorted normally in *ypt35*Δ cells in which endosomal localization of Vps13 is not visible. Moreover, in the *vps13-G718K N2428S* background, loss of *YPT35* appears to enhance the sorting defect. How can endosomal localization correlate with function in one instance and not the other, and how can *YPT35* enhance CPY sorting in the mutant background but be dispensable in the wild-type strain?

**Figure 7:**
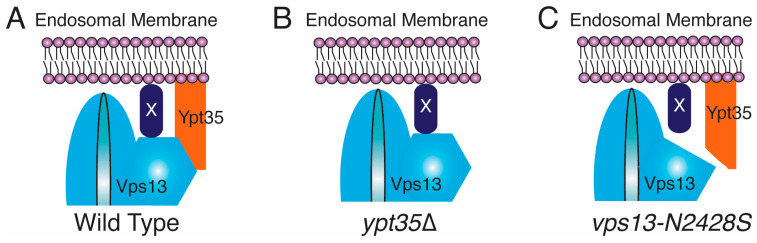
Two-adaptor model for Vps13 binding to the endosome. (**A**) In wild-type cells, Vps13 is stably recruited to the endosome by a strong interaction with *Ypt35* and a weak interaction with a second, hypothetical protein. Both proteins interact with the Vps13 VAB domain. Interaction with X is required for proper CPY sorting. (**B**) In *ypt35*Δ cells, only the weak interaction is present. This is sufficient for CPY sorting but not for a stable localization to the endosome. (**C**) In vps13-2428S cells, both interactions are disrupted and both localization and CPY sorting are lost.

One possible explanation is if the VAB domain of Vps13 actually binds to two ligands at the endosome (Figure 7). One of these “co-adaptors” would be *Ypt35*, which is required for stable endosomal localization, and the other co-adaptor is “protein X” that is essential for CPY sorting. We hypothesize that interactions between Vps13 and both co-adaptors, are weakened by the N2428S mutation. In *ypt35*Δ cells, Vps13 does not bind stably to endosomes, so no clear endosomal localization is seen, but it may still have a transient interaction with protein X that allows proper CPY sorting. In the *vps13-N2428S* mutant, both interactions are disrupted and so both stable localization at the endosome and CPY sorting are affected. Introduction of the G718K mutation partially restores binding to both ligands and so localization and function are rescued. However, in the presence of the N2428S mutation, interaction with protein X is not strong enough to allow function unless *Ypt35* is also present. Note that the VAB region of Vps13 has been found to bind to phosphatidylinositol-3-phosphate [8], so it is possible that X is a lipid rather than another protein.

While speculative, this “co-adaptor” model is consistent with other observations of Vps13 behavior. For example, two sporulation-specific proteins, Spo71 and Spo73, which interact with Vps13 are required for proper Vps13 activity at prospore membranes [22,42]. *SPO71* is essential for localization of Vps13 to the prospore membrane, but *SPO73* is also important for its function [13,42,43]. Thus, Spo71 and Spo73 could be prospore membrane co-adaptors. In mammalian cells, overexpression of the gene encoding the VPS13A partner protein XK recruits VPS13A to specific subdomains of the ER [27]. The XK protein still physically interacts with a VPS13A VAB domain mutant protein, VPS13A^W2460R^. However, overexpression of *XK* does not concentrate VPS13A^W2460R^ within the ER. These results suggest that localization to specific ER domains requires binding to both XK and a second partner protein, also consistent with the idea of co-adaptors [27].

Further investigation will be required to test this co-adaptor hypothesis. However, it is clear that the combination of missense alleles with *VPS13*-related pathologies identified in humans and the genetic tools available in the yeast provide a powerful model system in which to dissect the function of this protein family.

## 4. Materials and Methods

### 4.1. Yeast Strains and Media

Yeast strains used for this study are listed in Table 3. Unless otherwise mentioned, standard yeast media and genetic techniques were used [44]. Solid sporulation (SPO) medium contained 2% agar, 1% potassium acetate, 0.05% yeast extract and 0.05% glucose. Point mutations were introduced into the yeast *VPS13* gene using a two-step strategy described in [13]. As an example, to mutate the asparagine residue at position 2428 of *VPS13* to serine, the *SkHIS3* cassette was first amplified from pFA6a-SkHIS3MX6 [45] and used to replace the asparagine codon (nucleotides 7282 to 7284) in BY4741. The plasmid pRS425-Cas9-SkHIS3-381 [46], containing *LEU2*, *CAS9*, and a guide RNA directed at *SkHIS3* was then co-transformed into this strain along with a 73-nucleotide single-stranded rescue oligonucleotide that encoded the asparagine to serine mutation. Recombinational rescue of the Cas9-induced double strand break by the oligonucleotide resulted in introduction of the mutation into the chromosomal *VPS13* gene. Leu^+^ transformants were screened first for loss of growth on SD–His medium. The presence of the mutation was then confirmed by amplifying an ~2 kb fragment and sequencing a region of about 800 bp surrounding the mutation. This strategy was used to introduce all of the human cognate mutations into BY4741, as well as the *mmm1*Δ*::kanMX6* strain, JSP441. To generate JSP816 carrying *vps13-N2428S*^*GFP*, *SkHIS3MX6* was integrated between nucleotides 4079 and 4080 of the *VPS13* coding region in JSP757. A PCR fragment carrying the *GFP* coding sequence flanked by *VPS13* homology located 5′ and 3′ of the insertion site was co-transformed with pRS425-Cas9-SkHIS3-381 into JSBP757, resulting in the replacement of *SkHIS3* with *GFP* between amino acids 1360 and 1361. Glycine 718 was mutated to lysine in a strains JSP816 and JSP851 via the same CRISPR/Cas9 strategy described above, resulting in JSP832 (*vps13-G718K N2428S^GFP*) and JSP881 (*vps13-G718K N2428S RTN1*-*GFP*).

The diploids, JSYD11, JSYD12 and JSYD13, were created by mating BY4741, JSP757, or JSP816, respectively, to HI27 and then selecting for diploid cells on SD-Arg-Leu medium.

To delete the *YPT35* open reading frame (ORF), a PCR-based gene deletion method was used [45]. *YPT35* was deleted from JSP497 (*VPS13^GFP*) with either *kanMX6* (JSP871) or *hphMX4* (JSP861) and from JSP832 (*vps13-G718K N2428S^GFP*) with *hphMX4* (JSP856). The presence of the deletion alleles were confirmed by PCR.

The JSP863 and JSP864 haploids carrying *mRFP* fused to the 3′ end of *DID2* were constructed in several steps. First, *DID2*-*GFP*::*SkHIS3MX6* was amplified by PCR from the genome of GCY2 [47] and transformed into JSP816 and JSP832, selecting for His^+^ transformants (strains JSP857 and JSP858). *GFP::SkHIS3* was then replaced with *mRFP* using CRIPSR/Cas9. JSP857 and JSP858 were co-transformed with pRS425-Cas9-SkHIS3-381 and a healing fragment consisting of *mRFP* (amplified from pRS424-DTR1-mRFP [38]) flanked by sequences immediately 5′ and 3′ of the *DID2* stop codon. Integration of the *mRFP* tag was confirmed by observation of transformants containing red foci using epi-fluorescence microscopy.

To introduce the *RTN1*-*GFP*::*SkHIS3* allele into various haploids for the ER-phagy assay, the *RTN1*-*GFP::SkHIS3* coding region was amplified from GCY5 [47] and used to replace the *RTN1* ORF in KO2 (*atg40*Δ), KO1 (*vps13*Δ), RP201 (*vps13-L66P*), RP202 (*vps13-L1107P*), RP203 (*vps13-Y2702C*), JSP757 (*vps13-N2428S*), JSP758 (*vps13-R3015Q*), JSP770 (*vps13-N2216S*), and JSP549 (*vps13-G718K*) to create JSP845, JSP846, JSP847, JSP848, JSP849, JSP851, JSP852, JSP854, and JSP883, respectively. Integration of the GFP tag was confirmed by observation of green fluorescence in the transformants using epi-fluorescence microscopy. To generate JSP881 (*vps13-G718K N2428S RTN1*-*GFP*::*Skhis3*), the *SkHIS3* cassette was first mutated by transformation of JSP851 with pRS425-Cas9-SkHIS3-381 without a rescue oligonucleotide and screening for His^−^ transformants, followed by loss of the *LEU2* plasmid and the introduction of the G718K mutation using CRISPR-CAS9 as described above.

### 4.2. Mitochondrial Function Assay

A defect in mitochondrial homeostasis is manifested by the failure of a *vps13* mutant to grow in the absence of *MMM1* [12,13]. Haploid *vps13-X mmm1*Δ strains are viable in the presence of the *MMM1 URA3 CEN1* plasmid, pRS316-MMM1 [12,13]. Single colonies of these strains were grown to mid-log phase in YPD and serial ten-fold dilutions were spotted onto SD complete medium with or without 0.08% 5-FOA to select for cells that had lost the *MMM1* plasmid. Failure to grow after incubation at 30 °C for 4 to 7 days indicated a defect in mitochondrial homeostasis.

### 4.3. Vps13 Antibodies

Antibodies to yeast Vps13 were generated by Covance (Princeton, NJ, USA). A peptide corresponding to residues 1466 to 1482 of Vps13 (DNKHTELIPKSKNKEYQ) was used as the antigen to generate antisera in rabbits (Figure 2A). The specificity of the antibody was validated by the detection of a protein band at the predicted molecular weight > 250 kD that is absent in the *vps13*Δ (Figure 2B).

### 4.4. Western Blot Analysis

Five milliliters of exponentially growing cells (2~3 × 10^7^ cell/mL) were used for protein extraction. Cell pellets were resuspended in 5 mL of 5% trichloroacetic acid (TCA) and incubated at 4 °C for 10 min with agitation. After centrifugation at 4000 revolutions per min (RPM) for 5 min, the pellets were resuspended in 1 mL of room temperature acetone, transferred to 2 mL screw cap tubes, and placed in a microfuge at 13,000 RPM for 5 min. Supernatant was discarded and the pellets left to dry without caps for three hours. Pellets were resuspended in 200 µL lysis buffer (50 mM Tris/Cl pH 7.5, 1 mM EDTA, 2.75 mM Dithiothreitol, 1.1 mM Phenylmethylsulfonyl fluoride, Roche protease inhibitor cocktail; catalog # 04 693 159 001) and then broken up thoroughly using a pipet tip. Glass beads equivalent to a volume of 200 µL (BioSpec, Bartelsville, OK, USA) were added and the cells lysed using a FastPrep-24 cell lysis machine (MP Biomedical, Solon, OH, USA) at speed 6.5 for two pulses of 45 s each. After bead beating, 150 µL of 2 X sodium dodecyl sulfate (SDS) sample buffer (100 mM Tris-HCl pH 6.8, 4% SDS, 20% Glycerol, 0.2 mg/mL Bromophenol blue, 0.72 M 2-Mercaptoethanol) was added and the lysates incubated at 95 °C for 5 min. Soluble proteins were separated from cell debris and beads by centrifugation at 13,000 RPM for 5 min.

Proteins were fractionated using 7.5% SDS-polyacrylamide gels (200 volts, 45 min) and then transferred to polyvinylidene difluoride membranes (Merck Millipore Ltd., Burlington, MA, USA). Membranes were probed with anti-Vps13 antibodies at 1:500 dilution or anti-Arp7 antibodies (Santa Cruz Biotechnology, Santa Cruz, CA, USA) at 1:5000 dilution for 2 h at room temperature. Horseradish peroxidase (HRP) conjugated goat anti-rabbit IgG (Invitrogen, Carlsbad, CA, USA) and mouse anti-goat IgG (Santa Cruz Biotechnology, Santa Cruz, CA, USA) were used at 1:5000 dilution for Vps13 and Arp7, respectively. The chemiluminescent HRP activity was detected using a Clarity Western ECL Substrate kit (BioRad, Hercules, CA, USA) and a BioRad Chemidoc machine.

### 4.5. CPY Secretion Assay

The optical density (OD)_660_ of exponentially growing cultures was measured and used to adjust the cultures to a concentration of 1 × 10^7^ cells/mL. Serial dilutions were then spotted onto plates containing SD complete medium. Nitrocellulose membranes placed on top of the cells and the plates were incubated overnight at 30 °C. Membranes were then washed with Western blot washing buffer (10 mM Tris-HCl pH 7.5, 2.5 mM EDTA pH 8.0, 50 mM NaCl, 0.05% Tween-20) to remove attached cells and probed with monoclonal anti-CPY antibodies (Life Technologies, Carlsbad, CA, USA) at 1:1000 dilution, followed by the HRP conjugated goat anti-mouse IgG secondary antibody (cat. 31430, Invitrogen) at 1:5000 dilution.

### 4.6. ER-phagy Assay

ER-phagy was induced as previously described [14]. Briefly, cells were grown to exponential phase in YPD. Rapamycin was added to a final concentration of 200 ng/mL and the cultures were incubated for 16–18 h at 30 °C. The location of the vacuole was identified either by staining with 7-amino-4-chloromethylcoumarin (CMAC) (Molecular Probes, Eugene, OR, USA) or by differential interference contrast microscopy. ER-phagy was detected by the presence of Rtn1-GFP fluorescence in the vacuolar lumen by fluorescence microscopy.

### 4.7. Microscopy

Live cell imaging was performed on a Zeiss Imager.Z2 microscope (Carl Zeiss, Thornwood, NY, USA) with a Zeiss Axiocam 702 mono digital camera. ZEN 3.0 (Blue edition) software was used to acquire images.

To visualize the vacuolar lumen in live cells, CMAC was added to a final concentration of 100 µM to cells that were then incubated at room temperature for 15–30 min. Cells were washed with SD complete medium and observed by fluorescence microscopy.

## Figures and Tables

**Table 1:** Characterization of *VPS13* family disease alleles in yeast.

Human Gene	Mutation	Reference	Cognate Yeast Mutation	Protein Level	CPY Secretion	Growth in *mmm1*Δ	Sporulation	ER-Phagy
			*VPS13*	++	-	+	+	+
			*vps13*Δ	-	+++++	-	-	-
*VPS13A*	L67P	[35]	L66P	++	-	-	+	+
	A1095P	[26]	L1107P	+	++	-	+	+
	Y2721C	[5]	Y2702C	+++	-	+	+	+
*VPS13B*	N2993S	[3]	N2216S	+	-	+	+	+
*VPS13C*	W395C	[36]	W363C	+++	-	+	+	+
	A444P	[36]	F417P	++	-	+	+	+
	I2789T	[36]	L2229T	+++	-	+	+	+
*VPS13D*	L2900S	[2]	F1881S	+	-	+	+	ND
	N3521S	[2]	N2428S	+	++	+/−	+	-
	N4107I	[6]	D2896I	+++	-	+	+	ND
	A4210V	[6]	G2998V	+++	-	+	+	ND
	R4228Q	[6]	R3015Q	+++	-	+	+	+

ND = no data.

**Table 2:** Sporulation in *vps13-N2428S* compared to *VPS13*.

Strain	Genotype	Sporulation Efficiency (%) *
JSYD11	*vps13*Δ/*VPS13*	75 ± 5.9
JSYD2	*vps13*Δ/*vps13*Δ	0
JSYD12	*vps13*Δ/*vps13-N2428S*	71 ± 4.5

* Average percentage ± 1 S.D. *n* = 3, 300 cells scored per replicate.

**Table 3:** *S. cerevisiae* strains.

Strain	Genotype	Source
BY4741	*MAT***a***his3*Δ*0 leu2*Δ*0 met15*Δ*0 ura3*Δ*0 ho*	[48]
KO1	same as BY4741 except *vps13*Δ*::kanMX6*	[49]
KO2	same as BY4741 except *atg40*Δ*::kanMX6*	[49]
KO6	same as BY4741 except *ypt35*Δ*::kanMX6*	[49]
GCY5	same as BY4741 except *RTN1::GFP::SkHIS3MX6*	[47]
GCY2	same as BY4741 except *DID2::GFP::SkHIS3MX6*	[47]
RP201	same as BY4741 except *vps13-L66P*	[13]
RP202	same as BY4741 except *vps13-1107P*	[13]
RP203	same as BY4741 except *VPS13-Y2702C*	[13]
RP206	same as BY4741 except *VPS13-W363C*	this study
RP207	same as BY4741 except *VPS13-F417P*	this study
JSP729	same as BY4741 except *VPS13-L2229T*	this study
JSP757	same as BY4741 except *VPS13-N2428S*	this study
JSP758	same as BY4741 except *VPS13-R3015Q*	this study
JSP770	same as BY4741 except *VPS13-N2216S*	this study
JSP786	same as BY4741 except *VPS13-F1881S*	this study
JSP787	same as BY4741 except *VPS13-D2896I*	this study
JSP788	same as BY4741 except *VPS13-G2998V*	this study
JSP497	same as BY4741 except *VPS13^GFP*	[13]
JSP861	same as BY4741 except *VPS13^GFP ypt35*Δ*::hphMX4*	this study
JSP871	same as BY4741 except *VPS13^GFP ypt35*Δ*::kanMX6*	this study
JSP816	same as BY4741 except *vps13-N2428S^GFP*	this study
JSP857	same as BY4741 except *vps13-N2428S^GFP DID2::GFP::SkHIS3MX6*	this study
JSP863	same as BY4741 except *vps13-N2428S^GFP DID2::mRFP*	this study
JSP832	same as BY4741 except *vps13-G718K N2428S^GFP*	this study
JSP858	same as BY4741 except *vps13-G718K N2428S^GFP DID2::GFP::SkHIS3MX6*	this study
JSP864	same as BY4741 except *vps13-G718K N2428S^GFP DID2::mRFP*	this study
JSP856	same as BY4741 except *vps13-G718K N2428S^GFP ypt35*Δ*::hphMX4*	this study
JSP845	same as BY4741 except *atg40*Δ*::kanMX6 RTN1::GFP::SkHIS3MX6*	this study
JSP846	same as BY4741 except *vps13*Δ*::kanMX6 RTN1::GFP::SkHIS3MX6*	this study
JSP847	same as BY4741 except *vps13-L66P RTN1::GFP::SkHIS3MX6*	this study
JSP848	same as BY4741 except *vps13-L1107P RTN1::GFP::SkHIS3MX6*	this study
JSP849	same as BY4741 except *VPS13-Y2702C RTN1::GFP::SkHIS3MX6*	this study
JSP851	same as BY4741 except *vps13-N2428S RTN1::GFP::SkHIS3MX6*	this study
JSP852	same as BY4741 except *VPS13-R3015Q RTN1::GFP::SkHIS3MX6*	this study
JSP854	same as BY4741 except *VPS13-N2216S RTN1::GFP::SkHIS3MX6*	this study
JSP883	same as BY4741 except *vps13-G718K RTN1::GFP::SkHIS3MX6*	this study
JSP881	same as BY4741 except *vps13-G718K N2428S RTN1::GFP::SkHIS3MX6*	this study
JSP441	*MATα mmm1*Δ*::kanMX6 his3*Δ*SK leu2 trp1::hisG ura3/pRS316-MMM1*	[13]
JSP759	same as JSP441 except *vps13-N2428S*	this study
JSP443	*MAT***a***vps13*Δ*::SkHIS3MX6 mmm1*Δ*::kanMX6 leu2 trp1::hisG ura3 his3*Δ*SK/pRS316-MMM1*	[13]
BY4742	*MATα his3*Δ*0 leu2*Δ*0 lys2*Δ*0 ura3*Δ*0 ho*	[48]
JSP512	same as BY4742 except *VPS13^GFP DID2::mRFP*	[13]
JSP531	same as BY4742 except *vps13-G718K^GFP*	[13]
JSP549	same as BY4742 except *vps13-G718K*	[13]
HI27	*MAT*α *ura3 his3*Δ*SK trp1::hisG arg4-NspI lys2 ho*Δ*::LYS2 rme1::LEU2 leu2 vps13*Δ*::SkHIS3MX6*	[50]
JSYD1	*MAT***a** *leu2*Δ*0* *his3*Δ*0* *met15*Δ*0* *ura3*Δ*0* *TRP1* *ARG4* *LYS2* *MATα* *leu2* *his3*Δ*SK* *MET15* *ura3* *trp1::hisG* *arg4-NspI* *lys2* *ho* *RME1* *VPS13* *ho::LYS2* *rme1::LEU2* *VPS13*	[13]
JSYD2	same as JSYD1 only *vps13*Δ*::kanMX6/vps13*Δ*::SkHIS3MX6*	[13]
JSYD4	same as JSYD1 only *VPS13^GFP*/*vps13*Δ*::SkHIS3MX6*	[13]
JSYD11	same as JSYD1 only *VPS13*/*vps13*Δ*::SkHIS3MX6*	this study
JSYD12	same as JSYD1 only *vps13-N2428S/vps13*Δ*::SkHIS3MX6*	this study
JSYD13	same as JSYD1 only *vps13-N2428S^GFP/vps13*Δ*::SkHIS3MX6*	this study

“*^GFP*” indicates insertion of the gene encoding a green fluorescent protein after codon 1360 in the *VPS13* ORF.

## Data Availability

Data are contained within the article and Supplementary Materials.

## Supplementary Materials

The following are available online at https://www.mdpi.com/article/10.3390/ijms22126200/s1, Figure S1: Full blot of Western in Figure 2. Figure S2: Full blot of Western in Figure 3.
