# Supplementary figures and images for: Genetic Dissection of Vps13 Regulation in Yeast Using Disease Mutations from Human Orthologs
Source: Int J Mol Sci. 2021 Jun 8;22(12):6200. doi: 10.3390/ijms22126200 (PMC8229349; doi:10.3390/ijms22126200)

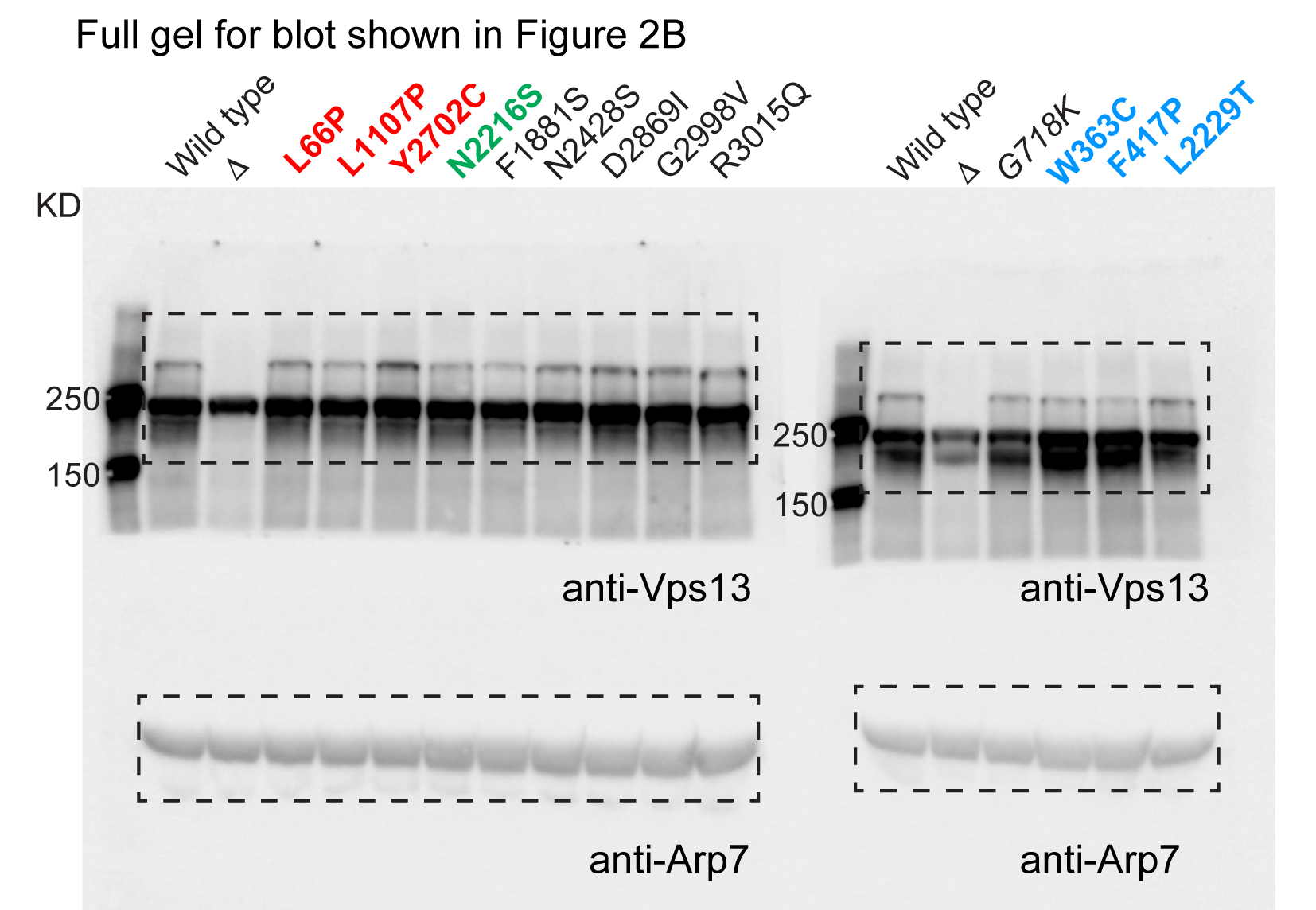

Supplement: Supplementary file 1 [file ijms-22-06200-s001.zip › Supplementary Figure S1.tif]

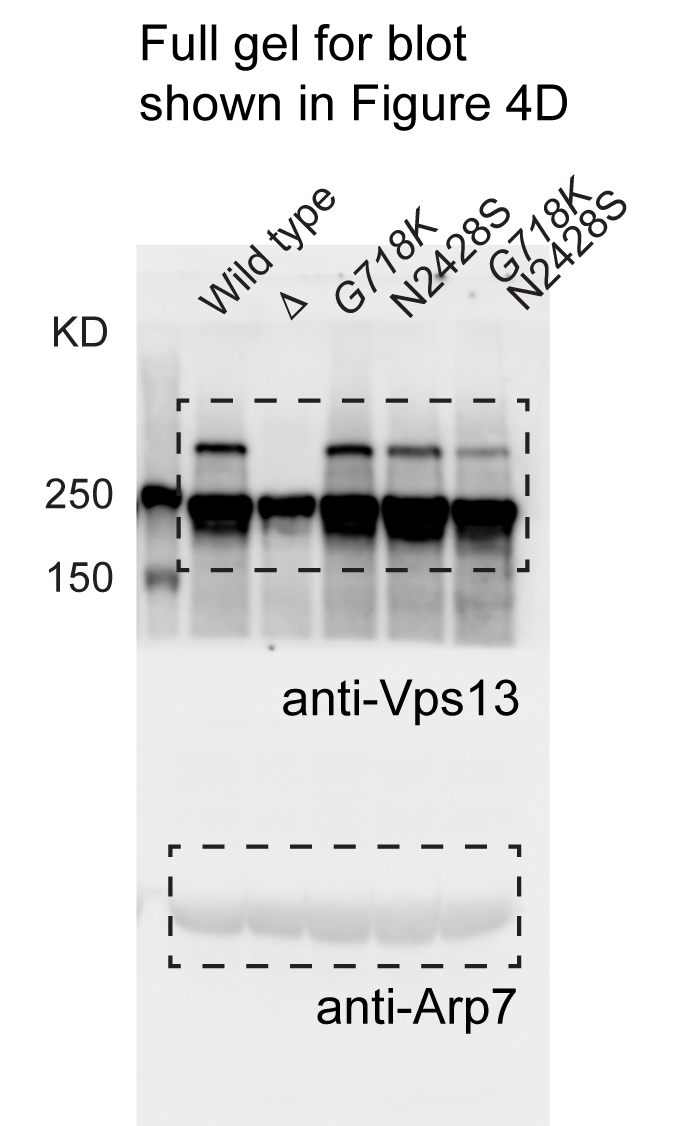

Supplement: Supplementary file 1 [file ijms-22-06200-s001.zip › Supplementary Figure S2.tif]
